# Recurrent nephrolithiasis and loss of kidney function: a cohort study

**DOI:** 10.1007/s11255-023-03463-x

**Published:** 2023-01-16

**Authors:** Rachel Yi Ping Tan, Nitesh N. Rao, Christopher M. Horwood, George Passaris, Rajiv Juneja

**Affiliations:** 1 Renal Unit, Flinders Medical Centre, Bedford Park, South Australia Australia; 2 College of Medicine and Public Health, Flinders University, Bedford Park, South Australia Australia; 3 Renal Unit, Lyell McEwin Hospital, Elizabeth Vale, South Australia Australia; 4 Adelaide Medical School, University of Adelaide, Adelaide, South Australia Australia; 5 Department of Clinical Epidemiology, Flinders Medical Centre, Bedford Park, South Australia Australia

**Keywords:** Nephrolithiasis, Renal calculi, Chronic kidney disease, GFR slope

## Abstract

**Purpose:**

To evaluate whether symptomatic recurrent nephrolithiasis leads to loss of kidney function.

**Methods:**

Adults who presented to the Emergency Department at least twice with symptomatic and radiologically confirmed nephrolithiasis were retrospectively recruited. Primary endpoint was the change in glomerular filtration rate (GFR) between baseline and at the time of data collection. Secondary endpoints include GFR slope defined as the mean rate of change in GFR from baseline to the end of the study period.

**Results:**

240 patients had recurrent symptomatic nephrolithiasis. Median follow-up was 5.4 years. The median age of first acute presentation was 51.6 years and the median baseline serum creatinine (bsCr) was 85.5 umol/l. 17.5% (*n* = 42) had worsening GFR, with the average change in GFR of − 8.64 ml/min/1.73 m^2^ per year. Four patients progressed to ESKD requiring haemodialysis. 14.5% (*n* = 35) had calcium oxalate stones. Univariate analysis showed older patients (*p* < 0.001), more symptomatic stone episodes (*p* < 0.001) and non-calcium-containing stones (*p* < 0.001) were strongly associated with deteriorating kidney function. Age (*p* = 0.002) and number of acute stone episodes (*p* = 0.011) were significant predictive factors when unadjusted to co-morbidities. Age (*p* = 0.018) was the only predictive factor of worsening GFR when adjusted for co-morbidities. Average mean GFR slope was − 2.83/min/1.73 m^2^ per year.

**Conclusions:**

Recurrent symptomatic nephrolithiasis is associated with loss of kidney function, in older patients, increased episodes of symptomatic nephrolithiasis and non-calcium-containing stones. Age is the only predictive factor for progression to chronic kidney disease in this subgroup.

**Supplementary Information:**

The online version contains supplementary material available at 10.1007/s11255-023-03463-x.

## Introduction

Nephrolithiasis is becoming more prevalent worldwide. In the U.S., almost 2 million outpatient visits were recorded with urolithiasis as the primary diagnosis in 2000 [1]. Collated data from Australian public hospital admissions between 2006 and 2007 showed an estimate annual incidence of 131 cases of upper urinary tract stone per 100,000 population [2]. Not only has nephrolithiasis been thought to increase the risk of cardiovascular events [3, 4], a recent meta-analysis showed that it may increase the risk of chronic kidney disease (CKD) regardless of patient population, and may be an important risk factor for end-stage kidney disease (ESKD) [5]. Therefore, *recurrent* nephrolithiasis can be said to pose a risk for ESKD although not commonly identified to be a primary cause of ESKD.

According to Vaughan et al., the risk of stone recurrence in 5 years ranged from 0.9 to 94% depending on risk factors, number of previous episodes, and years since the last episode [6]. A recent historical matched-cohort study with a mean follow-up of 12 years indicated 0.93% of stone formers compared to 0.36% of matched non-stone formers developed ESKD. The higher risk of ESKD in recurrent stone formers was evident before and after adjustment for co-morbidities [7].

This is the first study looking at the Australian cohort to determine the impact of recurrent symptomatic nephrolithiasis on kidney function.

## Methods

### Data source

Initial data were extracted using International Classification of Diseases, 10th revision (ICD-10) codes (N132, N135, N200, N201, N202, N209, N210, N211, N218, N219, N23) to identify Emergency Department (ED) presentations of nephrolithiasis between 1st January 2005 and 31st December 2017 at Flinders Medical Centre in South Australia. An interval of at least 1 month between codes represented a separate stone episode. De-identified data were collected between November and December 2019.

This study was approved by the Office for Research, Southern Adelaide Local Health Network, South Australia Health (Approval number 111.19). Informed consent was not required.

### Study design and patient selection

This was a retrospective cohort study to evaluate recurrent nephrolithiasis and loss of kidney function in the Australian cohort. Patients 18 years or older with at least two documented ED presentations with acute symptomatic nephrolithiasis, and stone visualised on imaging (computed tomography or ultrasound) or which required intervention during that 12-year study period were enrolled. We excluded pregnant patients and those on maintenance dialysis at baseline.

Radiology images and reports were reviewed to confirm nephrolithiasis and to classify the degree of obstruction if present. Where severity of obstruction was not reported, the grading system of hydronephrosis from Society of Foetal Urology was utilised. Where radiology imaging was not available, the operation report for any intervention of nephrolithiasis was reviewed to confirm obstruction.

### Variables collected

Baseline demographics at initial ED presentation were recorded. This consisted of age, gender, co-morbidities including diabetes, hypertension, obesity, ischaemic heart disease, inflammatory bowel disease, gout and previous gastrointestinal surgery. Gastrectomy, partial or total colectomy and appendicectomy were considered as previous gastrointestinal surgery. Underlying kidney conditions identified on imaging such as medullary sponge kidney, polycystic kidney disease, single functioning kidney or congenital abnormalities of the urinary system were documented.

All laboratory results were acquired from the same pathology service in South Australia. Modification of Diet in Renal Disease (MDRD) equation was used to calculate the estimated glomerular filtration rate (eGFR) until 2013. This was subsequently changed to Chronic Kidney Disease Epidemiology Collaboration (CKD-EPI) formula as it was validated in Aboriginals and Torres Strait Islanders population. Baseline serum creatinine (bsCr) and eGFR were defined as levels measured at least 1 month prior to initial ED presentation. However, the bsCr and eGFR from the first acute ED presentation were taken if there was no previous documentation of renal function. For missing eGFR values, MDRD equation was used to obtain an eGFR based on the serum creatinine and patient’s age at the time of the blood test.

The most recent sCr and eGFR were obtained at the end of data collection (December 2019). Patients who died or became dialysis dependent were recorded. Chronic kidney disease (CKD) was defined as eGFR < 60 ml/min/1.73 m^2^ for at least 3 months. All eGFR values then were recalculated using CKD-EPI formula [8] as indicated in the updated clinical practice guidelines [9] to measure the GFR slope.

The total number of acute ED presentations for recurrent nephrolithiasis for each patient was documented. Of those, the initial four ED presentations were assessed. For each presentation, two sCr measurements were obtained—the peak sCr during admission and last documented sCr on discharge. If there is no discharge sCr available for that stone episode, the same peak sCr value during admission is used as the discharge sCr measurement. Stone characteristics were also collected, including stone size based on diameter reported on imaging (in millimetres) with four subgroup ranges (less than 5 mm, 5–10 mm, 11–15 mm, more than 15 mm), location (renal pelvis, pelvic-ureteric junction, proximal ureter, mid-ureter, distal ureter, vesico-ureteric junction and bladder), and presence and severity of obstruction. All stone chemistries at any point during the study period were recorded. In patients where two stone compositions were detected, they were regarded as mixed stone formers. Metabolic workup at any point during the study period were also identified, including serum electrolytes, parathyroid hormone, vitamin D levels and 24-h urinary electrolytes.

### Statistical analysis

Statistical analyses were performed using Stata version 15 (Stata Corporation, College Station, Texas, USA). Continuous variables are expressed as mean ± standard deviation or median with interquartile ranges as appropriate. Categorical variables are expressed as counts with percentage. The primary outcome measured was the change between eGFR at baseline and at the end of data collection. Univariate analysis was performed using Pearson correlation test or two-sample *t* test to analyse eGFR change. Variables that were evaluated included age, number of nephrolithiasis episodes, cumulative stone diameter, degree of obstruction categorised between no obstruction and mild obstruction against moderate to severe obstruction, and stone composition categorised as those with or without calcium. Associations were summarised with corresponding 95% confidence intervals (CI). A multivariate linear regression model was developed, and this included the variables with significant associations from univariate analysis. The model was adjusted for co-morbidities including diabetes, hypertension, ischaemic heart disease, obesity, inflammatory bowel disease, gout, and previous bowel surgery. Associations were summarised with corresponding 95% confidence intervals (CI). All analyses with *p* value < 0.05 were considered statistically significant.

Mean decline in eGFR over time, also known as eGFR slope was measured. Slope was calculated as the coefficient of the best-fitted line estimated for the relationship between all eGFR values recalculated using CKD-EPI formula and time using simple linear least-squares regression analysis. A reduction in eGFR slope of at least 1.07 ± 0.42 ml/min/1.73 m^2^ per year [10] was considered clinically significant.

## Results

### Baseline characteristics

**Fig. 1:**
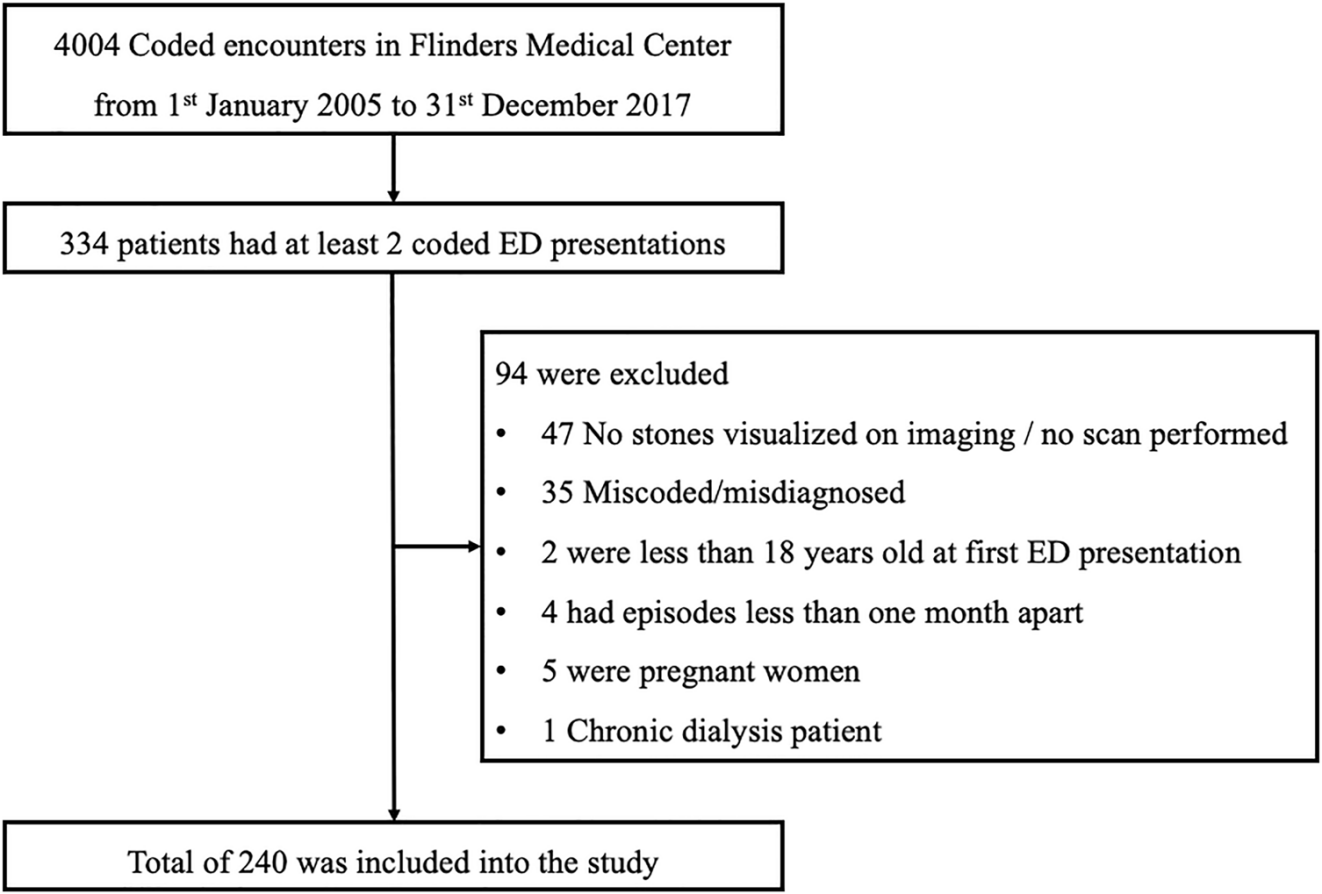
Sampling of the cohort presented to emergency department with at least two confirmed symptomatic episodes of nephrolithiasis from ICD-10 Coding

334 patients had at least 2 coded ED presentations. After chart review, 240 patients had confirmed recurrent symptomatic nephrolithiasis with a total 554 episodes incorporated in the final analysis (Fig. 1). Nine patients had more than four episodes documented throughout the study period—six patients had five presentations, and one patient each had six, seven and ten ED presentations, respectively.

Table 1 shows the baseline characteristics of the cohort studied. 196 (81.7%) patients were male, with a male to female ratio of 4.5:1. The median age at the first acute ED presentation with nephrolithiasis was 51.6 years (interquartile range 34.5–69). Hypertension was prevalent in 30%, followed by diabetes (19.2%) and ischaemic heart disease (12.9%). Six patients (2.5%) had inflammatory bowel disease. 11.7% had previous gastrointestinal surgery, with nine patients with a history of partial or total colectomy. The cohort studied had 11 patients with underlying renal disease; 7 patients had medullary sponge kidney, with the other 4 patients having polycystic kidney disease, single functioning kidney, horseshoe kidney and a duplex collecting system respectively. BsCr was 85.5 umol/l (interquartile range 73.4–100.8 umol/l) and the median baseline eGFR was 84 ± 28.3 (SD) ml/min/1.73 m^2^. 44.2% had CKD Stage 1. The percentage of patients with the same sCr on admission and discharge for each stone episode are 70%, 64.2%, 62.1% and 81.3% respectively (Table 2).

**Table 1:** Baseline characteristics of 240 patient cohort studied

Baseline demographics	
Males, *N* (%)	196 (81.7%)
Age at first ED presentation (years) (median, IQR)	51.6 (IQR 34.5–69)
Serum creatinine at baseline^a^ (umol/l) (median, IQR)	85.5 (IQR 73.4–100.8)
eGFR at baseline (ml/min/1.73 m^2^) (median ± SD)	84 ± 28.3
CKD staging classification^b^ (ml/min/1.73 m^2^), *N* (%)	
CKD Stage 1	106 (44.2%)
CKD Stage 2	109 (45.4%)
CKD Stage 3a	19 (7.9%)
CKD Stage 3b	3 (1.3%)
CKD Stage 4	1 (0.4%)
CKD Stage 5	2 (0.8%)
Co-morbidities^c^	
Diabetes, *N* (%)	46 (19.2%)
Hypertension, *N* (%)	72 (30%)
Ischaemic heart disease, *N* (%)	31(12.9%)
Obesity, *N* (%)	18 (7.5%)
Inflammatory bowel disease, *N* (%)	6 (2.5%)
Gout, *N* (%)	11 (4.6%)
Previous bowel surgery, *N* (%)	28 (11.7%)
Partial/total colectomy	9 (3.8%)
Appendicectomy^d^	18 (7.5%)
Gastrectomy	2 (0.8%)
Underlying renal disease (by imaging)	
Medullary sponge kidney, *N* (%)	7 (2.9%)
Polycystic kidney disease, *N* (%)	1 (0.4%)
Single functioning kidney, *N* (%) (not congenital)	1 (0.4%)
Congenital abnormalities, *N* (%)	
Horseshoe kidney	1 (0.4%)
Duplex collecting system	1 (0.4%)
Total number of ED presentations with nephrolithiasis	568 episodes

Abbreviations *ED* emergency department, *eGFR* estimated glomerular filtration rate. *CKD* chronic kidney disease; *IQR* interquartile range; *SD* standard deviation

^a^Baseline serum creatinine obtained from blood samples at least 1 month prior to initial ED presentation. 50 patients had bsCr and eGFR from the first acute ED presentation as there was no previous documentation of renal function

^b^CKD staging classification: (A) Stage 1: at least 90 ml/min/1.73 m^2^; (B) Stage 2: 60–89 ml/min/1.73 m^2^; (C) Stage 3a: 45–59 ml/min/1.73 m^2^; (D) Stage 3b: 30–44 ml/min/1.73 m^2^; (E) Stage 4: 15–29 ml/min/1.73 m^2^; (F) Stage 5: less than 15 ml/min/1.73 m^2^

^c^Patients may have more than one co-morbidity and at least two calculi size. ^d^ Only one patient had both appendicectomy and partial colectomy

**Table 2:** Breakdown of patients with documented with the same serum creatinine on admission and discharge for each stone episode

ED presentation	Total number of patients per episode	Number of patients with the same documented sCr on admission and discharge	Percentage of patients with the same documented sCr on admission and discharge (%)
1st episode	240	168	70
2nd episode	240	154	64.2
3rd episode	58	36	62.1
4th episode	16	13	81.3

Abbreviations: *sCr* serum creatinine

554 stones were detected during the study period. The weighted mean calculi size by diameter was 5.7 mm. They were most commonly located at the vesico-ureteric junction (23.3%), followed by distal ureter (17.7%) and proximal ureter (17.3%). 45.1% of all episodes had mild hydronephrosis. Only 24 episodes (4.3%) had severe hydronephrosis. Of the 118 patients where stone composition was available, 14.6% had calcium oxalate stones, followed by mixed calcium oxalate and calcium phosphate stones (12.5%) and pure uric acid stones (9.6%) (Table 3).

**Table 3:** Baseline stone characteristics

Stone characteristics	
Calculi size based on diameter^a^	
Mean renal calculi size (mm)	5.7 (min 0, max 21)
Number of calculi size ranging	
i. Less than 5 mm	247 (44.6%)
ii. 5–10 mm	264 (47.7%)
iii. 11–15 mm	30 (5.4%)
iv. More than 15 mm	13 (2.3%)
Calculi location^a^, *N* (%)	
Renal pelvis	87 (15.7%)
Pelvic-ureteric junction	57 (10.3%)
Proximal ureter	96 (17.3%)
Mid-ureter	61 (11.0%)
Distal ureter	98 (17.7%)
Vesico-ureteric junction	129 (23.3%)
Bladder	26 (4.7%)
Obstruction^a^, *N* (%)	
No obstruction	118 (21.3%)
Hydroureter only	85 (15.3%)
Mild hydronephrosis	250 (45.1%)
Moderate hydronephrosis	77 (13.9%)
Severe hydronephrosis	24 (4.3%)
Calculi composition^b^, *N* (%)	
Calcium oxalate	35 (14.6%)
Calcium phosphate	6 (2.5%)
Uric acid	23 (9.6%)
Cysteine	1 (0.4%)
Struvite (magnesium ammonium phosphate)	1 (0.4%)
Calcium oxalate and calcium phosphate	30 (12.5%)
Calcium oxalate and uric acid	16 (6.7%)
Calcium oxalate and ammonium phosphate	4 (1.7%)
Calcium phosphate and ammonium phosphate	2 (0.8%)
Unknown	122 (50.8%)

^a^Total of 554 stones were detected on renal imaging (CT/US). 8 imaging reports did not comment on size and were assumed as zero. This included three episodes of staghorn calculi which may underestimate mean calculi size. Patients may also have at least two calculi size but only the largest stone size was accounted for

^b^Stone composition collected for 240 patients

PTH and vitamin D levels were available for 32 (13.3%) and 64 (26.7%) patients, respectively. 34 (14.2%) patients had 24-h urinary electrolytes performed. Supplementary Table S1 provides the number of patients whose 24-h urine electrolyte collection was available with and without their stone chemistries.

Median follow-up was 5.4 years. Four patients progressed to end-stage kidney failure requiring haemodialysis. During the study period, 11 patients died—5 due to malignancy, 1 due to hospital acquired pneumonia, and 5 where the cause was unknown (died as outpatient).

### Primary outcome

42 of the 184 patients had a worsening eGFR (17.5%). When adjusting the change in eGFR in relation to time between baseline eGFR and at the end of data collection, the average change in eGFR was − 1.32 ml/min/1.73 m^2^ per year. In patients with a reduction of eGFR, the average change in eGFR was − 8.64 ml/min/1.73 m^2^ per year. 14 had an improvement in eGFR (5.8%) but 8 of these 14 patients had unknown baseline renal function prior to their first ED presentation.

### Secondary outcomes

**Fig. 2:**
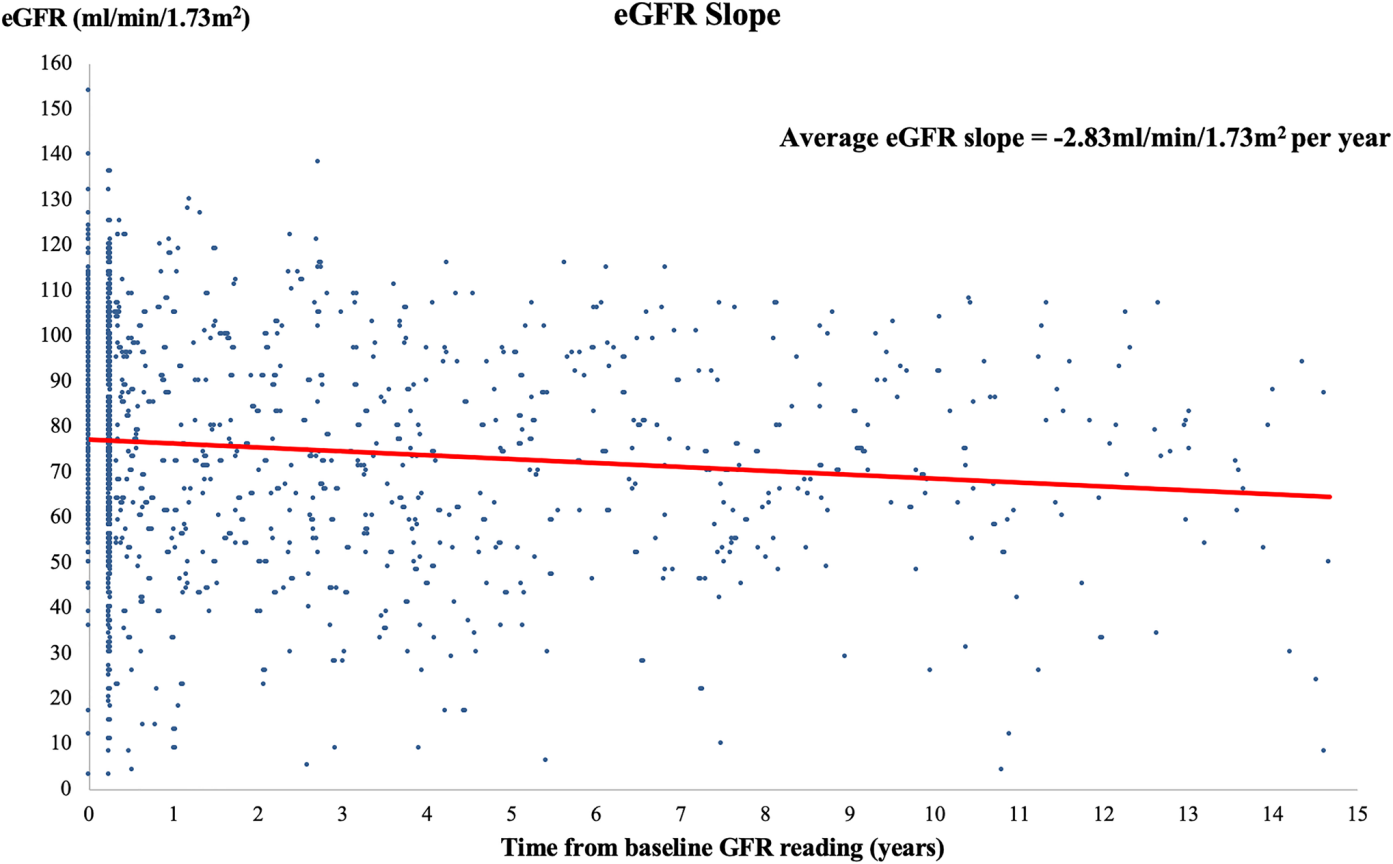
Scatterplot demonstrating the average eGFR slope for 240 patients calculated from serum creatinine at baseline to the end of data collection (December 2019). Each point (blue) represents an eGFR value. The solid (red) line is the average eGFR slope which is − 2.83 ml/min/1.73 m^2^ per year. The mean eGFR slope in healthy subjects is − 1.07 ± 0.42 ml/min/1.73 m^2^ per year [10]

Table 4 shows the variables analysed as potential predictors of deteriorating kidney function. Univariate analyses for all 240 patients revealed a reduction of eGFR that was significantly associated with age (*p* < 0.001), number of symptomatic episodes of nephrolithiasis (*p* < 0.001) and cumulative stone size diameter (*p* = 0.004). There was no significant association of reduction of eGFR with degree of obstruction (*p* = 0.089). Univariate analysis for 118 patients where stone composition was available demonstrated that non-calcium-containing nephrolithiasis was significantly associated with a reduction in eGFR (*p* < 0.001). A multivariate linear regression model demonstrated older patients (*p* < 0.001) and increased episodes of symptomatic nephrolithiasis (*p* = 0.011) were significant predictors for a reduction in eGFR unadjusted for co-morbidities. Cumulative stone size was not a significant predictive factor for reduced eGFR (*p* = 0.894). Only age was a significant predictor for reduction in eGFR when adjusted for all co-morbidities (*p* = 0.018). The average eGFR slope is ‒ 2.83 ± 14.5 ml/min/1.73 m^2^ per year (Fig. 2).

**Table 4:** Univariate and multivariate analyses to determine predictors for deteriorating kidney function in recurrent symptomatic nephrolithiasis

Variables	Univariate	Analysis	Multivariate	Analysis ^c^	Multivariate	Analysis ^d^
	Correlation coefficient	*p* value	B (95% CI)	*p* value	B (95% CI)	*p* value
Age^a^	− 0.21	** < 0.001**	− 0.13 (− 0.21 to − 0.05)	**0.002**	− 0.10 (− 0.19 to − 0.02)	**0.018**
Number of nephrolithiasis episodes^a^	− 0.24	** < 0.001**	− 2.15 (− 3.80 to − 0.49)	**0.011**	− 1.15 (− 2.74 to 0.44)	0.155
Cumulative stone size diameter^a^	− 0.18	**0.004**	0.03 (− 0.35 to 0.40)	0.894	− 0.09 (− 0.44 to 0.28)	0.635
Highest degree of obstruction^b^		0.089	− 0.85 (− 3.19 to 1.50)	0.476	0.05 (− 2.20 to 2.30)	0.965
Stone composition (*N* = 118)^b^		** < 0.001**	N/A		N/A	

*p* value < 0.05 is statistically significant and highlighted in bold

Abbreviations: *95% CI* 95% confidence interval

^a^Univariate analysis performed using Pearson correlation test, rounded to the nearest two decimal points

^b^Univariate analysis performed using two-sample *t* test, rounded to the nearest two decimal points

^c^Multivariate analysis unadjusted for co-morbidities

^d^Multivariate analysis adjusted for co-morbidities. Co-morbidities evaluated in multivariate analysis include diabetes, hypertension, ischaemic heart disease, obesity, inflammatory bowel disease, gout, and previous bowel surgery

## Discussion

There is a risk of developing CKD in patients with recurrent symptomatic and radiographic evidence of nephrolithiasis who presents to the Emergency Department. In patients with a decline in kidney function, the average change in eGFR was − 8.64 ml/min/1.73 m^2^ per year. We also analysed the eGFR slope which is now emerging as a surrogate clinical endpoint for progression of CKD [11]. This is the first known study to use this method for patients with recurrent nephrolithiasis. A longitudinal study by Baba et al. was conducted in 72,521 healthy subjects to evaluate decline in kidney function over an 8-year period [10]. This study had a mean (± SD) eGFR of 83.7 ± 14.7 ml/min/1.73 m^2^ and a mean rate of eGFR decline of 1.07 ± 0.42 ml/min/1.73 m^2^ per year. Our study showed a higher mean rate of eGFR decline of 2.83 ± 14.5 ml/min/1.73 m^2^ per year which further confirms progression of CKD in this patient group.

Our results were also consistent with previous study findings despite different definitions for a recurrent nephrolithiasis episode. A cohort study by Alexander et al. compared 23,706 stone formers with 3,065,488 non-stone formers using ICD-9 and ICD-10 derived from registry data from the Alberta Kidney Disease Network database and the Northern and Southern Alberta Renal Programs [12]. Their definition for a separate stone episode was an interval of at least 1 year between claims and adjusted co-morbidities. With a median follow-up of 11 years, stone formers had an increased risk of new stage 3b–5 chronic kidney disease (HR 1.74; CI 1.61–1.88) and ESKD (HR 2.16; 95% CI 1.79–2.62) compared to those without history of nephrolithiasis. This was significantly higher in those with more than one kidney stone episode, similar to that observed in our study. Interestingly, the risk increased with the number of previous stone episodes and female stone formers were at higher risk for ESKD compared with male stone formers (HR of 3.36 vs 1.87; *p* = 0.003).

Another cross-sectional, case–control study of 195 adult patients conducted in Iceland [13] defined recurrent nephrolithiasis as episodes occurring at least 6 months apart with a clinical stone event as acute flank pain associated with haematuria and/or stone detection by imaging studies or patient-reported stone passage. They found patients with recurrent nephrolithiasis had significantly lower kidney function after adjustment for body size, hypertension, diabetes and cardiovascular disease compared to the control group with no history of nephrolithiasis.

We also found that age was a significant predictor for reduction in eGFR when adjusted for co-morbidities. This is supported by a study conducted by Haley et al. which compared kidney function of incident symptomatic patients with their first episode of nephrolithiasis and age- and sex-matched controls [14]. They found that in symptomatic first-time stone formers, serum creatinine resolves after an initial rise in serum creatinine.

Furthermore, we observed that a higher number of symptomatic episodes of nephrolithiasis were significantly associated with a greater likelihood in the loss of kidney function, but it was not a predictive indicator when adjusted for hypertension, diabetes, ischaemic heart disease, obesity, inflammatory bowel disease and gout.

The pathophysiology behind recurrent nephrolithiasis and CKD is likely multi-faceted. Despite patients with nephrolithiasis having co-morbidities associated with CKD, such as diabetes, hypertension and gout [15], there is increasing literature showing specific nephrolithiasis-induced kidney injury. From a microscopic level, animal models with unilateral ureteral obstruction revealed parenchymal damage with tubulointerstitial inflammation and fibrosis mediated by up-regulation in transforming growth factor-beta and tumour necrosis factor-alpha. Macrophages were present as early as 4 h after onset of obstruction [16]. Renal parenchymal damage is also likely sustained in first episode symptomatic stone formers where they had a persistently higher cystatin C levels and proteinuria [14], both of which are poor prognostic markers for ESKD [17, 18], despite a resolution of their transient elevation of serum creatinine before and after the stone event. We can, therefore, hypothesise that with recurrent symptomatic stone episodes, there is recurrent tubulointerstitial inflammation and scarring, leading to progressive loss of kidney function. It is worth noting that although the degree of obstruction was not significantly associated with deteriorating kidney function in our study, it is plausible that a higher degree of obstruction could have led to earlier intervention which may have halted the inflammatory process and, therefore, prevented permanent renal parenchymal damage.

Similarly, stone-specific factors such as stone composition [19] and stone burden [20] have also been implicated as risk factors for CKD. A retrospective study of 1918 patients in Taiwan where stone chemistry and baseline eGFR were evaluated showed that eGFR was significantly lower in patients with non-calcium-containing nephrolithiasis (struvite and uric acid) than in patients with calcium-containing nephrolithiasis [19]. This is in keeping with our study findings that non-calcium-containing nephrolithiasis was significantly associated with reduced kidney function in patients with recurrent nephrolithiasis. Stone composition was not included in the multivariate analysis due to less than half of the patients having stone composition available.

We also found a significant association between cumulative stone size and deterioration of kidney function, but it was not considered a predictive factor for CKD. This is in contrast to a prospective study of 97 external shockwave lithotripsy patients in Iran. They found in individuals with cumulative stone size of less than 20 mm, it was an independent predictor for CKD; each 1 mm increase in cumulative stone size was associated with a 20% increased risk of CKD [21].

Another important consideration of CKD progression includes medications such as nonsteroidal anti-inflammatory drugs (NSAIDs) for pain relief which was not collected in our study. Whilst there is well-reported evidence of NSAIDs-related acute kidney injury, a recent review showed the risk of CKD progression from NSAIDs to be small and relative to cumulative dosing. They have recommended that there is no increased risk of progression of CKD in patients with CKD stage 1–3 in the absence of risk factors such as being more than 65 years old, having coronary artery disease and concomitant analgesic use [22]. This confounding factor may overestimate our findings but likely in a small proportion given the prolonged study period.

We take note of the 14 patients who had an improvement of their renal function, for which over half did not have a baseline eGFR. As a result, their baseline eGFR was considered as their eGFR at the time of their first ED presentation. This may reflect a false improvement of their renal function due to the lack of previous eGFR readings. We also observed the lack of 24-h urinary electrolytes within our cohort. This could be due to two main factors: data collection was confined to one pathology service which was approved by our ethics committee, or it was not ordered by the treating physician.

This study has several strengths. To date, this is the first known study which examines recurrent nephrolithiasis in an Australian population. It also has a reasonably long follow-up period to evaluate for CKD. Second, we only selected patients who had both clinical symptoms and radiological diagnosis or at least requiring intervention, which minimised the risk of including asymptomatic and incidental stone formers. Finally, we evaluated composition of nephrolithiasis associated with CKD which have not been previously reported.

Conversely, this study has several limitations that should be addressed. First, due to the retrospective nature of our study, there is a paucity of unmeasured confounding factors such as collection of baseline medications like NSAIDS contributing to a reduction in kidney function, and lifestyle factors which can increase risk of nephrolithiasis. We also found the lack of key variables like 24-h urinary electrolytes being performed which may further assist in patient’s care. Second, we had 50 patients who did not have a baseline eGFR prior to their first ED presentation with symptomatic nephrolithiasis. This may underestimate the change of our primary outcome. Finally, we could not consider medications and lifestyle factors which may increase the risk of nephrolithiasis due to the retrospective nature of our study. Thus, we cannot exclude the possibility of some unmeasured confounding factors in the association between recurrent symptomatic nephrolithiasis and the risk of loss of kidney function in the long term.

In this cohort with recurrent symptomatic and radiologically confirmed nephrolithiasis, the risk of CKD is positively associated with the increased number of ED presentations for nephrolithiasis. The risk of CKD is also impacted by being older, having non-calcium-containing stones and cumulatively larger stones with age being a predictive factor for CKD progression. Our study highlights the importance for physicians to advocate for stone prevention strategies to reduce nephrolithiasis and consequently preserve kidney function, especially in those patients with a first episode of nephrolithiasis.

## Supplementary Information

Below is the link to the electronic supplementary material.

Supplementary file1 (DOCX 30 KB)

## Data Availability

Data collection sheet has been stored in a password-protected computer server only accessible to investigators.
