# Supplementary material for: Recurrent nephrolithiasis and loss of kidney function: a cohort study
Source: Int Urol Nephrol. 2023 Jan 16;55(6):1539–47. doi: 10.1007/s11255-023-03463-x (PMC10185628; doi:10.1007/s11255-023-03463-x)
Supplement: Supplementary file 1 — Supplementary file1 (DOCX 30 KB) [file 11255_2023_3463_MOESM1_ESM.docx]

Supplementary Table S1: Proportion of patients with the availability of 24-hour urinary electrolytes collection and stone chemistry.

| 24-hr urinary electrolytes collected  Stone Chemistry | Yes | No |
| --- | --- | --- |
| Available | 23 | 95 |
| Not Available | 11 | 110 |
